# Hysterosalpingographic findings in women with genital tuberculosis

**Published:** 2015-05

**Authors:** Donya Farrokh, Parvaneh Layegh, Monavvar Afzalaghaee, Mohaddeseh Mohammadi, Yalda Fallah Rastegar

**Affiliations:** 1 *Surgical Oncology Research Center, Mashhad University of Medical Sciences, Mashhad, Iran.*; 2 *Department of Radiology, Imam Reza Hospital, Faculty of Medicine, Mashhad University of Medical Sciences, Mashhad, Iran.*; 3 *Department of Biostatistics and Epidemiology, Faculty of Health Medicine, Mashhad University of Medical Sciences, Mashhad, Iran.*

**Keywords:** *Tuberculosis*, *Female genital*, *Hysterosalpingography*, *Salpingitis*, *Infertility*, *Fallopian tube diseases*

## Abstract

**Background:**

Genital tuberculosis (TB) is an important cause of infertility in the developing countries, where hysterosalpingography (HSG) remains an initial diagnostic procedure in the evaluation of tubal and peritoneal factors leading to infertility.

**Objective:**

The aim of this study was to determine the HSG findings of genital TB in infertile women.

**Materials and Methods:**

We retrospectively reviewed HSG findings in 20 women with genital tuberculosis. HSG was performed in these women as part of infertility work up over 5 years. The other diagnostic procedures used included endometrial curettage and biopsy, histological examination, culture, laparoscopy, hysteroscopy and polymerase chain reaction.

**Results:**

The mean age of the participants was 30.5±8 years. All women had clinical history of infertility for at least 4 years. Women presented with pelvic abdominal pain (30-35%) and menstrual disturbances (20-25%). Reviewing 20 cases of female genital TB were encountered various presentations on HSG.

**Conclusion:**

HSG is an invaluable procedure in suggesting the diagnosis of genital TB in patients being investigated for infertility.

## Introduction

Genital tuberculosis (TB) is an important cause of health problem and infertility in many developing countries, where hysterosalpingography (HSG) remains the initial diagnostic procedure in the evaluation of tubal, uterine cavity, and peritoneal factors leading to infertility (1). A review of the literature demonstrated that the highest incidence of genital TB is in Africa and Asia (especially Pakistan, India, and Afghanistan) (2). The real incidence of genital TB in Iran is still unknown. It is difficult to determine the actual incidence of genital TB because there are no constant clinical symptoms and majority of the cases are discovered incidentally, through infertility work up. 

According to geographic location, socioeconomic statements and public health conditions, TB incidence is varying. The disease is caused by mycobacterium tuberculosis and is almost always acquired by hematogeneous spread from an extra genital source, such as pulmonary or abdominal TB. The fallopian tubes are most commonly affected. The endometrium is the second most common site affected by genital TB after the fallopian tubes and is involved in 50-60% of all cases. Involvement of the ovary and cervix is rare (ovary in 20% of cases and cervix in 5% of patients) (3). Genital TB tends to manifest as infertility, menstrual irregularities, amenorrhea, vaginal discharge, and post-menopausal bleeding, unexplained fever over a prolonged period, weight loss and/or chronic pelvic or lower abdominal pain. The disease is often associated with pelviperitoneal adhesions (the Fitz-Hugh Curtis syndromes) (4). 

Hysteroscopy is the gold standard for diagnosing uterine adhesions, distortion of the uterine cavity, and tubal Ostia, but HSG is the choice imaging method for evaluation the abnormalities of the fallopian tubes in patients with genital TB. HSG is a helpful imaging modality for evaluating the internal architecture of the female genital tract and is the most invaluable procedure in the assessment of tubal factor infertility in developing countries. The aim of this research is to illustrate the various HSG appearance in 20 women, who had underwent initial evaluation of infertility by HSG and were found to have genital TB. 

These cases were proved either by curettage and biopsy of the endometrium, detection of tubercles and adhesions at laparoscopy or laparotomy, and acid-fast bacilli culture. Tubal involvement may lead to tubal deformities such as irregular contour, diverticular, out pouching, beaded or pipe stem appearance, tubal occlusion, hydrosalpinx and peritubal adhesions. TB causes uterine cavity adhesions and distortions, sometimes with sever synechiae and complete obliteration of the uterine cavity (5-7).

## Materials and methods

This was a retrospective study of 20 women who had a proven genital TB between September 2002 and March 2010 at Imam Reza Hospital. The study was approved by the ethics committee of Mashhad University of Medical Sciences, Mashhad, Iran. The medical records of patients were reviewed for data collection. 

All women underwent HSG as an initial investigation for primary and secondary infertility. The type and duration of the infertility, personal history, and clinical symptoms (including menstrual irregularities, abdominal and pelvic pain, fever and general symptoms) were recorded for all women. The HSG was performed between the 8^th^ and 11^th^ day of the menstrual cycle. Water soluble contrast medium was introduced using a canola placed in the cervical canal under aseptic condition. Films were taken with the woman in supine anteroposterior projection and oblique views were done when necessary. 

An average of 8-15 ml of contrast medium was administered for each woman exept in two cases in whom up to 25 ml contrast media was injected because of the incomplete filling of the uterine cavity. Genital TB was proved in these women by several methods such as endometrial curettage or biopsy for acid-fact bacilli, histopathologic examination of the status of the endometrium and presence of tuberculosis granulomas, hysteroscopy and laparoscopy. The women included in this study had all undergone HSG to investigate tubal patency as an initial infertility work up and who were later found to have genital tuberculosis. 

The patients were grouped according to their kind of infertility (primary and secondary). Clinical data were analyzed and HSG findings were evaluated. Films were re-reported by a radiologist/ gynecologist. All HSG were checked for unilateral and bilateral spillage of contrast medium into the peritoneal cavity, and abnormalities in the outline of uterine cavity (such as irregular cavity, intraluminal filling defect, shrunken cavity, and the presence of synechia or T-shaped cavity), and deformity of fallopian tubes (such as tubal block, hydrosalpinx, beaded appearance pipe stem appearance, tobacco pouch appearance, and golf club appearance).

**Statistical analysis**

The data were expressed in the form of mean±SD, and Chi-square test was conducted for data analysis using SPSS software (Statistical Package for Social Sciences, version 13.0, SPSS Inc, Chicago, Illinois, USA). p˂0.05 were considered as significant differences.

## Results

The mean age of the participants was 30.5±8 years. The socioeconomic background of the women showed they belong to a poor (80%) or lower middle (20%) class. The original of 4 (20%) women in this study was from Afghanistan and the others were Iranian. 60% of these women had primary infertility and 40% had secondary infertility. Genital tuberculosis was diagnosed by various methods in these women. HSG was abnormal in all of these cases and the various abnormal findings at HSG in the uterus and fallopian tubes were evaluated. 

A normal uterine cavity was observed in 11 (55%) women, an irregular cavity in 3 (15%) women, an Irregular intrauterine filling defect in 2 (10%), T-Shaped cavity in 1 (5%) women, a small shrunken cavity in 2 (10%) women and synechiae in 3 (15%) women. Four women with uterine abnormality had more than one abnormal finding at HSG.

The right tubal abnormality was seen in 12 women. The results of Chi-square test showed that the right fallopian tube was significantly more involved than the left side (p<0.05). 

The most common tubal abnormalities were tubal occlusion and hydrosalpinx which were more common on the right fallopian tube. Complete tubal occlusion was observed in 12 (60%) patients and hydrosalpinx in 6 (30%) patients. Hydrosalpinx was associated with complete tubal blockage in 4 cases and in 2 (10%) patients there was incomplete blockage with a mild peritoneal spillage of contrast medium.

The fallopian tubes were patent in 30% of cases (right side in 10% and left side in 20%). In patients with tubal occlusion the most common site of tubal blockage was at the mid tubal which was detected in 8 cases. The Chi-square test showed that mid tubal blockage significantly occurred than other locations (p<0.05). 

The irregularities of the fallopian tubes was noted in 3 (15%) of the patients and ampullary diverticula was seen in 2 (5%) of the patients. A rigid pipe stem appearance was seen in 1 (5%) of cases and beaded appearance in 2 (10%) patients. There was evidence of pelvic peritoneal adhesions in 6 (30%) of the patients.

Ovarian calcification was seen in one patient and we did not observe tubal calcification in any patients. HSG demonstrated venous and lymphatic intravasation of contrast media in the pelvic vessels in 4 (20%) patients.

Chest x-ray was performed for all patients after the diagnosis of genital TB. The x-ray demonstrated the radiographic findings of pulmonary TB in 2 (10%) of the patients.

## Discussion

TB remains the most common cause of mortality from infectious disease. Every year around 7 million people worldwide develop TB; of these 2 million will die, with 90% of death occurring in low income countries (2). In recent years the incidence of genital TB has been steadily decreased in developed countries, but it remains a major health problem in many developing countries in Africa and Asia. The previously reported incidence of genital TB that causing infertility was 13% in 1979 (5). Genital tuberculosis is almost always acquired by haematogeneous spread from an extra genital source (3, 6). 

There is direct relationship between prevalence of genital TB and pulmonary TB. About 75% of cases with active genital TB have a normal chest x-ray, so a normal chest x-ray cannot exclude the diagnosis of genital TB (8, 9). 

The primary focus of genital TB is the fallopian tubes, which are usually affected bilaterally but not symmetrically (6-8). The fallopian tubes are the most common affected genital organs, followed by endometrium, ovary and cervix (6, 8, 9). In spite of significant advances in imaging modalities such as ultrasound, computed tomography (CT) and magnetic resonance imaging (MRI), HSG remains the gold standard imaging procedure in evaluating the internal architecture of the female genital tract especially fallopian tubes. Also it is a helpful procedure in the diagnosis of genital TB (8, 10). 

HSG is a safe, simple, inexpensive and rapid imaging test which can show the internal surface of uterine cavity and fallopian tubes. Some authors have substituted laparoscopy for HSG due to the higher false positive results in the diagnosis of tubal block and its failure to detect mild to moderate peritoneal adhesions (6, 7). 

In low developing countries, it is not cost effective to perform laparoscopy for all infertile women, and HSG continue to use as a preliminary procedure for investigating the abnormalities of the uterus and fallopian tubes in women presented with infertility. Laparoscopy is better in evaluating extra tubal intraperitoneal pathology such as peritubal adhesion and HSG is better in detecting intrauterine abnormalities and tubal patency (11). 

Pathologically, in genital TB, fallopian tubes are thickened and caseous ulceration and also diverticular out pouching of both the isthmus and ampulla may be seen (9, 12, 13). Entire tube become encased in heavy connective scar tissue and then developed a beaded or a rigid pipe stem appearance (6, 7). Tubal occlusion usually occurs in the isthmus and also ampullary portion of the fallopian tubes (6, 14, 15).Tubal obstruction may cause hydrosalpinx (6, 9). Tubal TB may spread to the endometrium in one half of patients. So a negative culture from endometrial curettage cannot exclude the diagnosis of genital TB (6-8).

**Figure 1:**
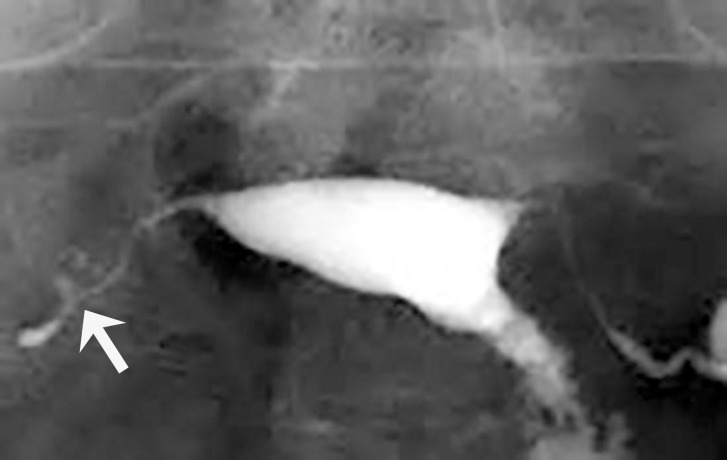
A 28-year-old woman with genital tuberculosis. Hysterosalpingogram shows irregular contour and diverticular out pouching at the right fallopian tube associated with tubal occlusion (arrow). Uterine cavity shows a normal appearance

The various abnormalities on HSG depend on the site of the involvement and the severity of the disease. The presence of causeous ulceration of the mucosa of the fallopian tube, irregular tubal contour and diverticular out pouching around the tube may cause a tufted appearance Isthmic diverticula may resemble salpingitis isthmica nodosa but in tubal TB, diverticular out pouching are larger, asymmetric, and are not usually restricted to the isthmic portion of the tube as compared with those of classic salpingitis isthmica nodosa (7) (Figure 1). 

**Figure 2:**
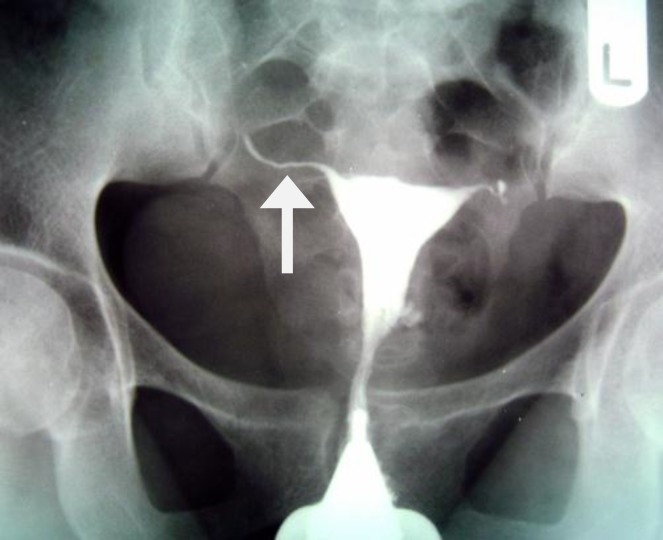
A 38-year-old woman with genital tuberculosis. Hysterosalpingogram shows bilateral tubal occlusion at the isthmic portion of the fallopian tubes (arrows). Uterine cavity has normal size and shape

**Figure 3:**
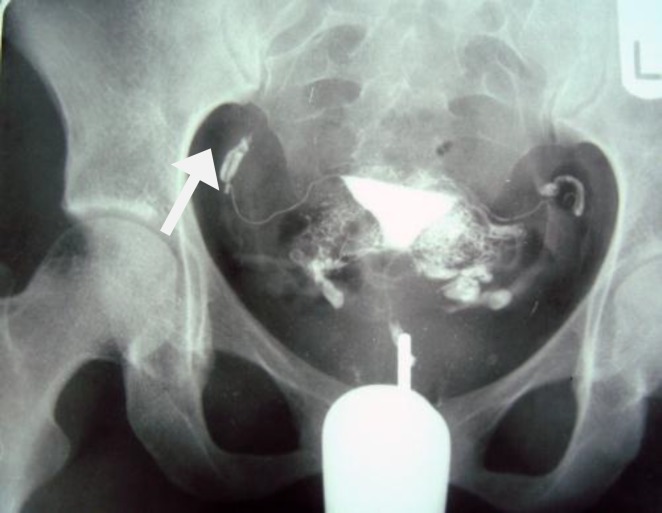
A 38-year-old woman with genital tuberculosis. Hysterosalpingogram shows bilateral tubal occlusion in the distal portion (arrows). Uterine cavity is normal. Intravasation of contrast medium into the pelvic vein is evident

**Figure 4:**
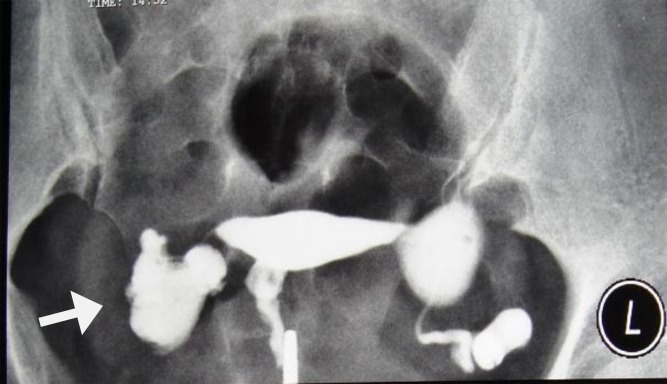
A 38-year-old woman with genital tuberculosis. Hysterosalpingogram shows terminal saculation and occlusion of both fallopian tubes causing hydrosalpinx (arrows). Uterine cavity has normal appearance.

**Figure 5:**
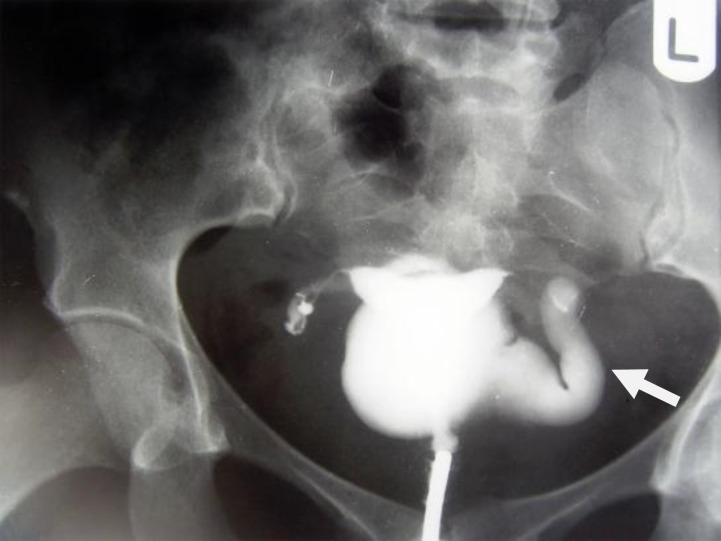
A 34-year-old woman with genital tuberculosis. Hysterosalpingogram shows (Floral appearance). Twisted hydrosalpinx resemble a floral appearance at the left side (arrow). Right tube is occluded. Uterine cavity has a normal size and shape

Tubal occlusion is the most common HSG findings in genital TB, which usually occurs in the isthmus and ampulla (Figure 2, 3) (6, 7). Corneal obstruction is not a common finding in genital TB on HSG. Tubal occlusion at the isthmic or fimberiated end of the fallopian tube with collection of serous or clear fluid may produce a sausage shaped dilatation of the tube, which initially is a pyosalpinx that change to hydrosalpinx (7). Hydrosalpinx is usually moderate or slight (Figure 4). Dilatation of the fallopian tube may cause a "Golf club appearance" (6, 7). Twisting of the hydrosalpinx may result in a floral pattern "Floral hydrosalpinx" (Figure 5). Intraluminal scaring can rise to a cobblestone pattern which is an effective radiographic sign of intraluminal adhesion in hydrosalpinx and associated with concern of infertility (7, 8). Although fallopian tubes are usually blocked in women with genital TB, but they may be patent in 37% of patients (16). 

**Figure 6:**
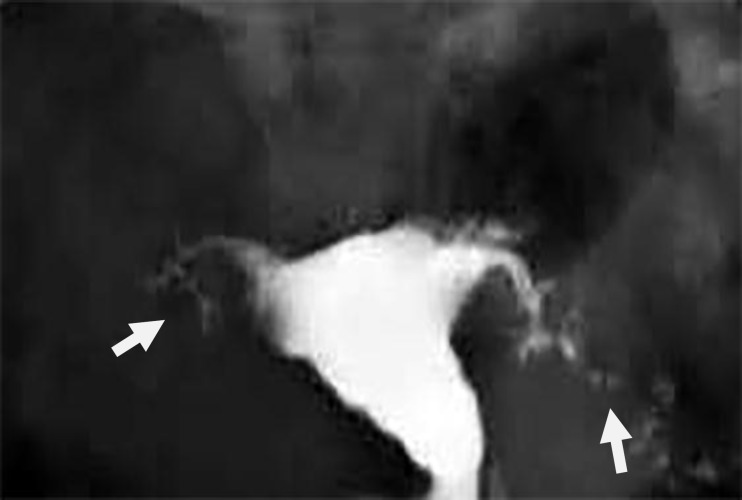
A 30-year-old-woman with genital tuberculosis. Hysterosalpingogram shows a beaded appearance at the right and left fallopian tubes associated with bilateral tubal occlusion (arrows). There is mild irregularity of the uterine cavity outline

**Figure 7:**
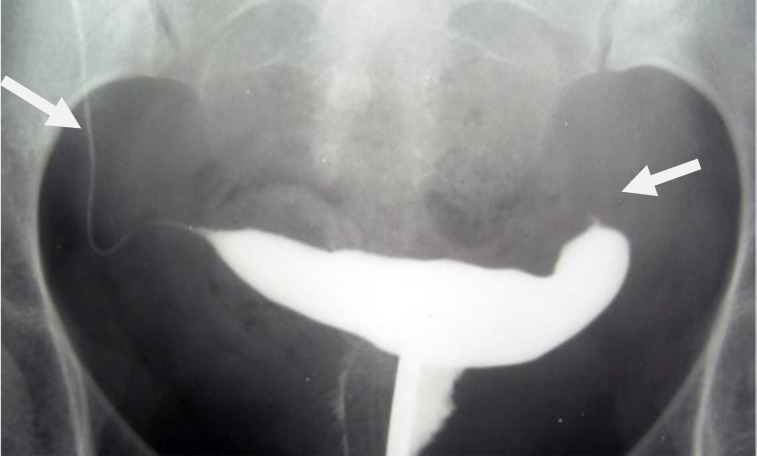
A 26-year-old woman with genital tuberculosis. HSG shows a large uterine cavity due to a fibromatouse uterus, obstruction of the corneal portion of the left tube and pipe stem appearance of the right tube (arrows).

In Sharma’s series the most common site of tubal obstruction was corneal (57%) (11). Multiple stricture along the fallopian tube can form and lead to a beaded appearances on HSG (Figure 6). Significant scaring of the fallopian tube may cause a rigid pipe stem appearance (6-8) (Figure 7). 

**Figure 8:**
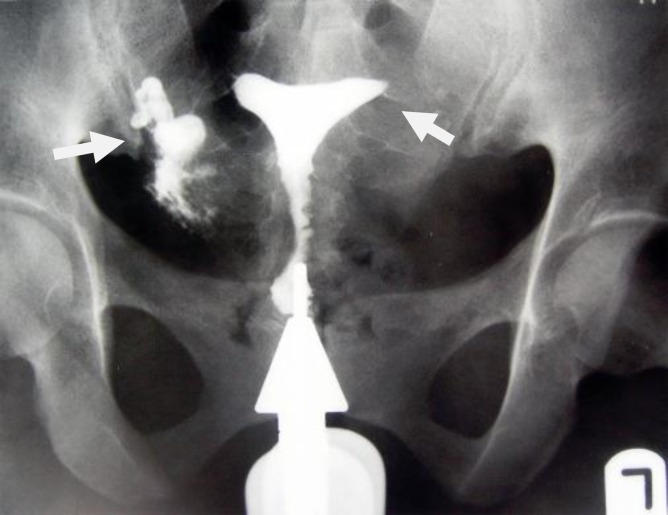
A 28-year-old woman with genital tuberculosis. Hysterosalpingogram shows terminal sacculation in right fallopian tube due to the peritoneal adhesion, Left tube is occluded (arrows). Uterine cavity is normal.

**Figure 9:**
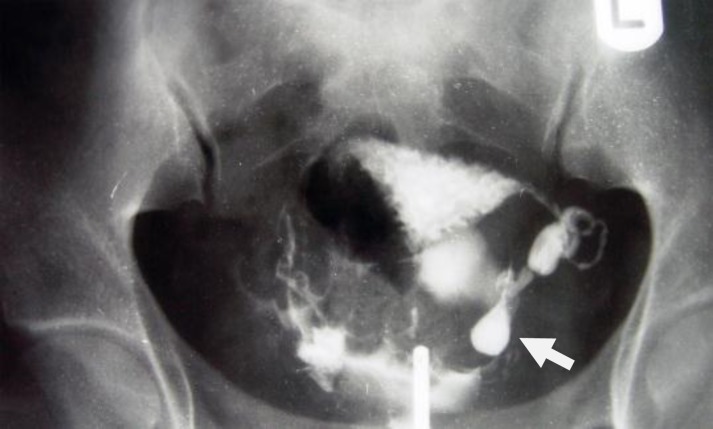
A 28-year-old woman with genital tuberculosis. HSG demonstrates evidence of peritoneal adhesion associated with a mild locculated spill on the left side (arrow). The contour of the uterine cavity is irregular

Pelvic peritoneal adhesions may happen in genital TB with peritoneal involvement and may disrupt the anatomical relationship between the fallopian tube and the ovary (7, 10, 12).The presence of a convoluted or corkscrew fallopian tube, peritoneal halo, tubal fixation and loculation of contrast medium in pelvic cavity is suggestive of peritoneal adhesion (6, 7) (Figure 8, 9). Sever peritoneal adhesions may lead to visualization of irregular septation with "Criss-cross spill pattern" (7, 9). The presence of a convoluted or corkscrew fallopian tube, peritoneal halo, tubal fixation and loculation of contrast medium in pelvic cavity is suggestive of peritoneal adhesions (6, 7). 

**Figure 10:**
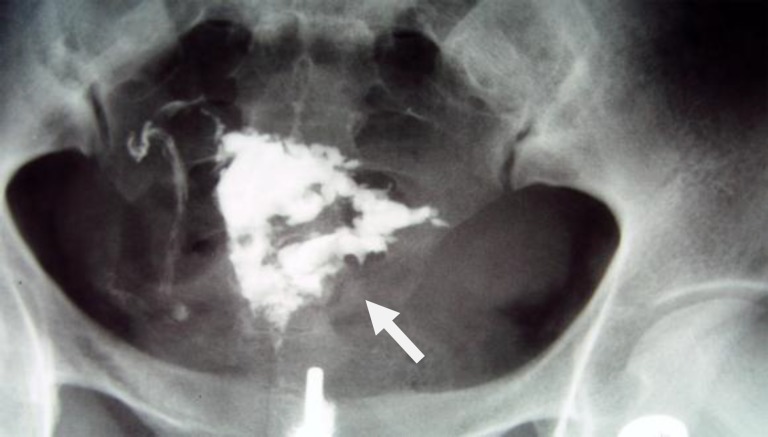
A 40-year-old woman with genital tuberculosis. Hysterosalpingogram shows multiple irregular intraluminal filling defect, irregularity and indentation of the uterine cavity contour due to synechiae resemble a denticulate uterus (arrow). Both fallopian tubes are occluded.

**Figure 11:**
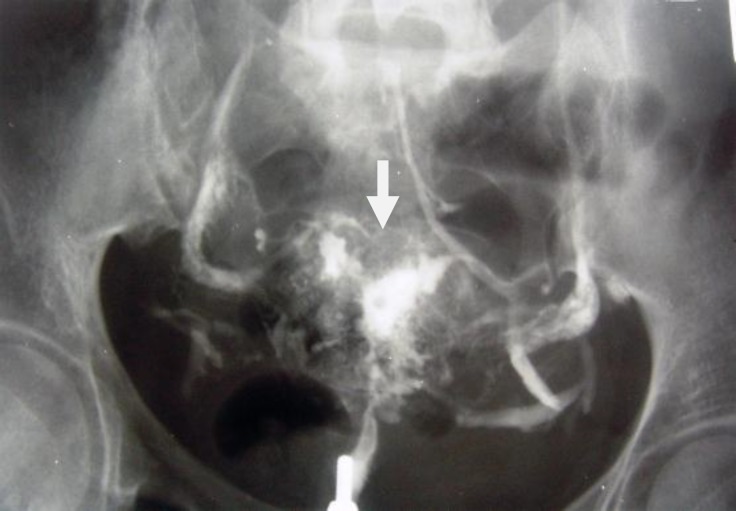
A 40-year-old woman with genital tuberculosis. Hysterosalpingogram shows irregular contour of the uterine cavity with diminished capacity resembling a trifoliate like appearance (arrow). Obstruction of the fallopian tubes and intravasation of contrast medium are seen.

**Figure 12:**
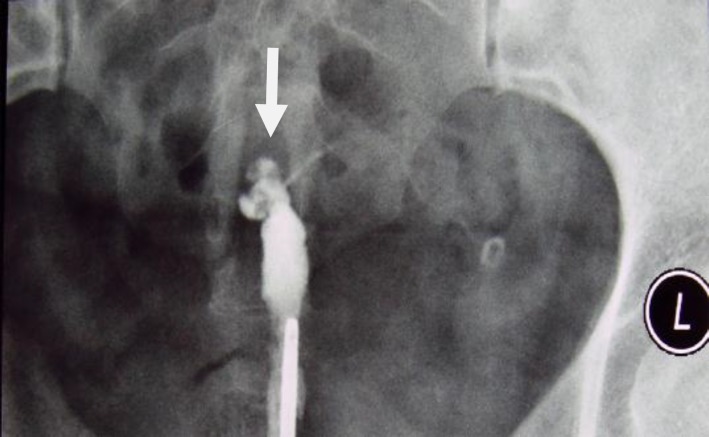
A-38-year-old woman with genital tuberculosis. Hysterosalpingogram shows small contracted uterine cavity with irregular contour and bilateral tubal occlusion (arrow).

Plain film of the pelvis may show fallopian tube or ovarian calcification which should be differentiated from calcified lymph nodes, calcified uterine fibromas, pelvic phlebolitis and calcification in ovarian dermoids (9, 13). In our series ovarian calcification was evident in only one (5%) of the patient at HSG. Endometrial tuberculosis may shows a non-specific appearance on HSG, like evidences of endometritis, including intrauterine adhesions, synechiae, irregularities and distortion of the uterine contour, asymmetric uterine cavity, venous and lymphatic intravasation and specific appearance such as T-shaped uterus and pesudounicornate appearance (6, 8). The synechiae and intracavitary adhesions in genital TB are usually irregular, angulated and stellate shaped with well-defined borders (Figure 10, 11). Scaring of the uterine cavity may cause conversion of the triangular uterine cavity into a T-shaped uterus (6, 9, 13, 15). Unilateral obliteration of the uterine cavity associated with unilateral scar may cause a pseudounicornate appearance. True unicorn ate uterus can be differentiated from pseudo unicorn ate deformity in genital TB by showing a smooth couture, and normal ippsilateral fallopian tube (9). Long duration of tubercular infection, extensive destruction of endomatrium and myometrium followed by fibrosis and complete obliteration of the uterine cavity may occur as the "Netar Syndrome" (9). Sever adhesions and fibrosis with complete obstruction of uterine cavity can lead to a gloves finger deformity that only cervical canal and a small portion of uterine cavity may be seen on HSG (Figure 12). Asymmetric small sized, shrunken uterine cavity at HSG is usually due to TB (6, 8, 9). Other radiographic patterns of uterine involvement include the formation of a "Colar stude abscess" which is pathogonomonic for tubercular lesion. This finding usually present as a collection of contrast medium that has a narrow neck and a brooder base is away from the uterine cavity (9). Blind ending sinus tract or fistulization to adjacent bowel are rare HSG findings of genital TB (3, 8).

**Figure 13:**
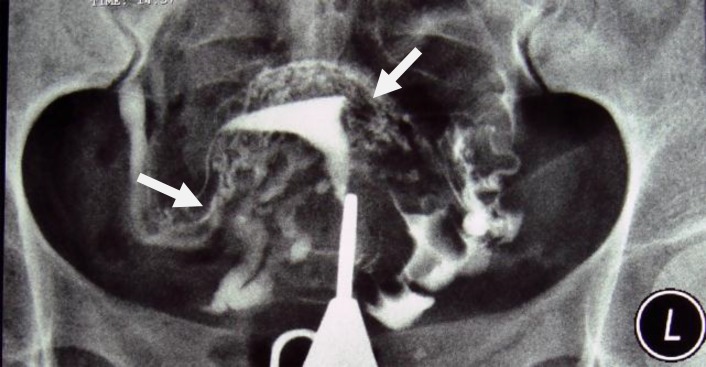
A 30-year-old woman with genital tuberculosis. Hysterosalpingogram demonstrates a bilateral tubal occlusion and intravasation of contrast medium in the uterine wall and pelvis (arrows). Uterine cavity appears normal

Venous and lymphatic intravasation is a good finding on HSG suggesting endometrial TB, and is due to the progressive destruction and ulceration of the endometrium (11, 14) (Figure 13). This feature is not specific for TB and may be seen in HSG done early in the menstrual cycle, shortly after endometrial instrumentation and in any pathology causing obstruction to the flow of contrast media (9, 13-15) (Figure 8). 

Cervical tuberculosis is rare due to the natural resistance of stratified epithelium of the ectocervix to bacterial penetration. Cervical involvement is usually secondary to tuberculous salpingitis and endometritis. On HSG ulceration of the cervical mucosa leads to an irregular contours and diverticular out pouching with a feathery appearance. The other features include cervical adhesions, which causes serrated and irregular end cervical canal (8, 9, 17).

In Sharma’s study 70 infertile women who underwent HSG to investigate infertility and subsequently diagnosed with genital TB were evaluated in 2007 in India and normal tubes were seen in 22% of patients, beaded appearance in 6%, and tobacco pouch appearance in 5%, hydrosalpinx in 39% and the other tubal abnormalities in 4% (9). Chavhan *et al* reviewed 37 cases of genital TB and tubal irregularity was seen in 6 (16%) at HSG and ampullary diverticula in 3 (8%), tubal block in 3 (9%) of patients, block with hydrosalpinx in 12 (32%), a rigid pipe stem appearance in 9 (24%) and beaded appearance in 6 (16%) patients (6). In comparison with the Sharma’s and Charkha’s studies, the incidence of tubal abnormalities such as pipe stem and beade appearance was lower in our study. Also hydrosalpinx was more common in Sharma’s series then our series.

In Sharma’s study normal uterine cavity was observed in 57.1% of women, an irregular cavity in 16.5%, a shrunken cavity in 2.8%, an irregular filling defect in 18.5% and synechiae in 17% of patients (9). Chavhan *et al* observed synechiae and intrauterine adhesions in 16% of women and venous and lymphatic intravasation in 27% in their series (6). Sharma *et al* found similar results with intrauterine synechiae in 17% and venous and lymphatic intravasation in 25% of women with genital TB (9). Venous and lymphatic intravasation was seen in 4 (20%) patients. We found similar results with intrauterine synechiae in 15% and interavasation in 20% of our patients. Genital tuberculosis is asymptomatic in many patients which makes it a silent cause of infertility (16, 18). HSG can help to diagnose these asymptomatic cases of genital TB (18, 19). 

There are multiple HSG findings for diagnosis of tubal TB given by various authors (6, 13, 20, 21);

1. Calcified lymph nodes or irregular calcification in the pelvic (adnexal) area.

2. Tubal obstruction between the isthmus and the ampullary portion of the fallopian tube.

3. Multiple strictures in the fallopian tube or beaded appearance.

4. Tobacco pouch appearance secondary to an everted fimbria with a patent orifice. 

5. Pipe stem appearance due to striated rigid contour of the fallopian tube.

6. Golf club appearance with slight or moderate dilatation of the ampullary portion of the fallopian tube. 

7. Rosette type appearance secondary to multiple small diverticular like out pouching surrounding the ampulla produced by caseous ulceration. 

8. Endometrial adhesion, synechiae, and or deformity or obliteration of the uterine cavity in the absence of clinical history of curettage or uteri surgery.

## Conclusion

In conclusion, HSG remains to be as an initial and valuable diagnostic procedure to evaluate infertile women. Even though the diagnosis of genital tuberculosis is possible by the demonstration of mycobacterium in the female genital system the characteristic appearances on HSG are reliable indicators, suggesting a diagnosis of genital TB in patients undergoing investigation for infertility and can identify the tubal and uterine involvement in most patients.
